# Supplementary material for: GIFT: An ImageJ macro for automated fiber diameter quantification
Source: PLoS One. 2022 Oct 3;17(10):e0275528. doi: 10.1371/journal.pone.0275528 (PMC9529089; doi:10.1371/journal.pone.0275528)
Supplement: S1 File — (DOCX) [file pone.0275528.s001.docx]

**Supporting Information**

**S1 Table: Summary of GIFT macro changes.** The GIFT macro was developed based on the published GIFT method [14], which was extensively reworked to create a macro with better functionality and which produced better outcomes. The main changes and upgrades are summarized.

| **Feature** | **Original Version** | **GIFT macro** |
| --- | --- | --- |
| **Thresholding method** | Constant pixel grayscale value used to threshold all images | A percent-based pixel value is calculated for each image individually |
| **Gaussian fit** | Fit performed in separate software (OriginPro) | Fit performed in ImageJ |
| **Morphological Filter** | Filtering step performed in separate software (OriginPro) | Morphological filtering (Erosion and Dilation) performed in ImageJ using the installed “Morphological Filter” Plugin. |
| **User interface** | No graphical user interface (GUI), parameters changed manually | A single GUI allows for control of all image-processing parameters. Additional control over file-saving options and fiber orientation analysis are available. |
| **Output files** | Manual file saving only | After analysis, the program prompts users to select the preferred file type for saving the data. Raw and summary data files are saved. Analysis parameters included in the summary data. |
| **Interactive image analysis** | Cropping and scale measurement determined manually in ImageJ before analysis | Users can interactively select the crop size, threshold level and scale bar length based on the first image in a batch during batch processing. These values are then automatically applied to all images in the batch. |


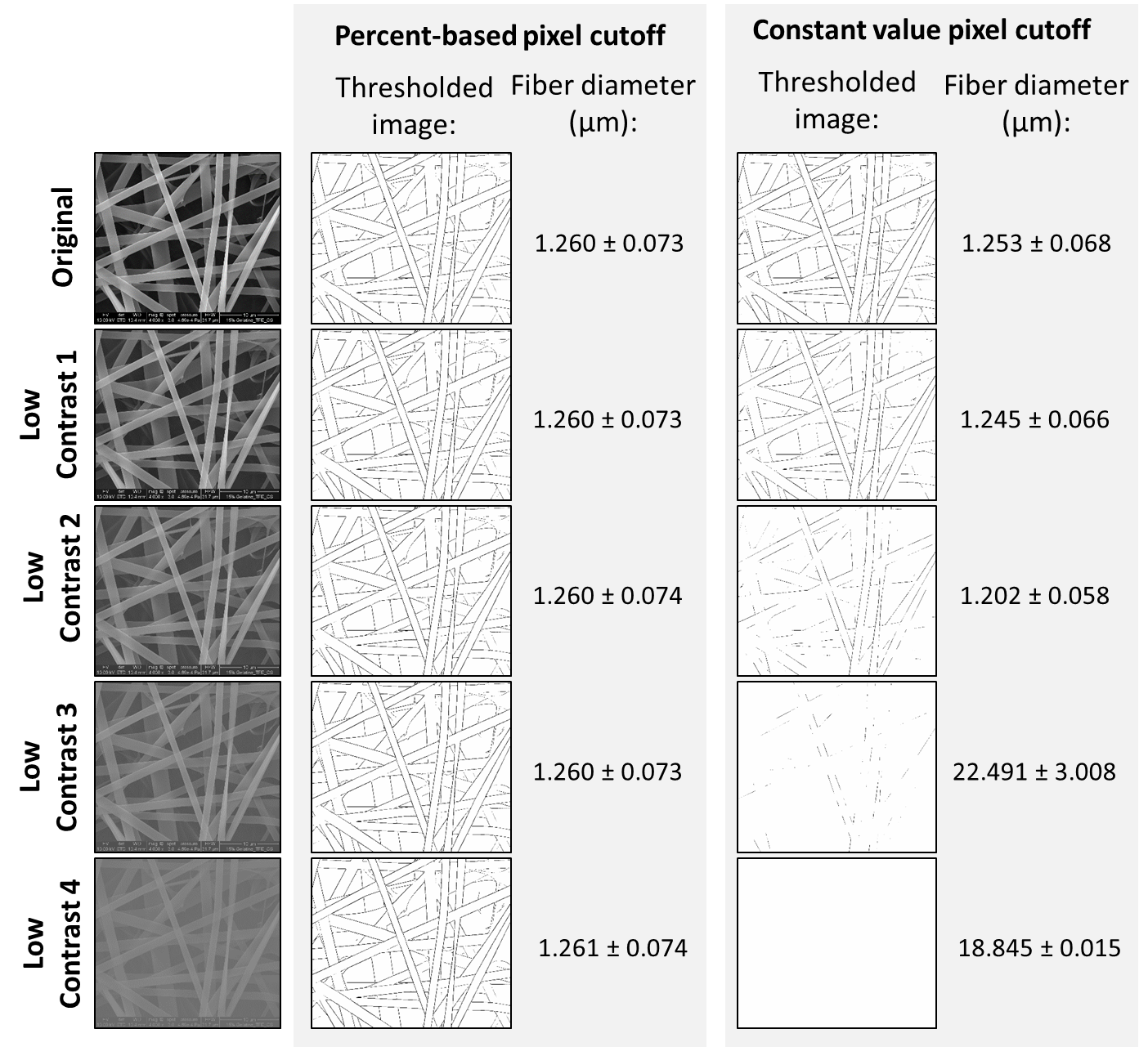


**S1 Fig. Testing thresholding methods with low contrast images.** A set of test images was made using the 15% gelatin SEM fiber image. The original image was adjusted in ImageJ to have progressively lower contrast. These images were analyzed with the GIFT macro using either percent-based thresholding or a constant thresholding value. For the percent based analysis, the threshold cutoff was determined so that 5% of darkest pixels remained after thresholding and this was calculated for each image. For the constant value thresholding analysis, a cutoff grayscale value of 200 was used for all images. The original images are shown in the left-hand column. The results of the thresholding image analysis step and the resulting average fiber diameter measured in each case are shown for both thresholding methods. It is clear that the adaptive, percent-based method is more consistent when presented with a batch of images with variable contrast.


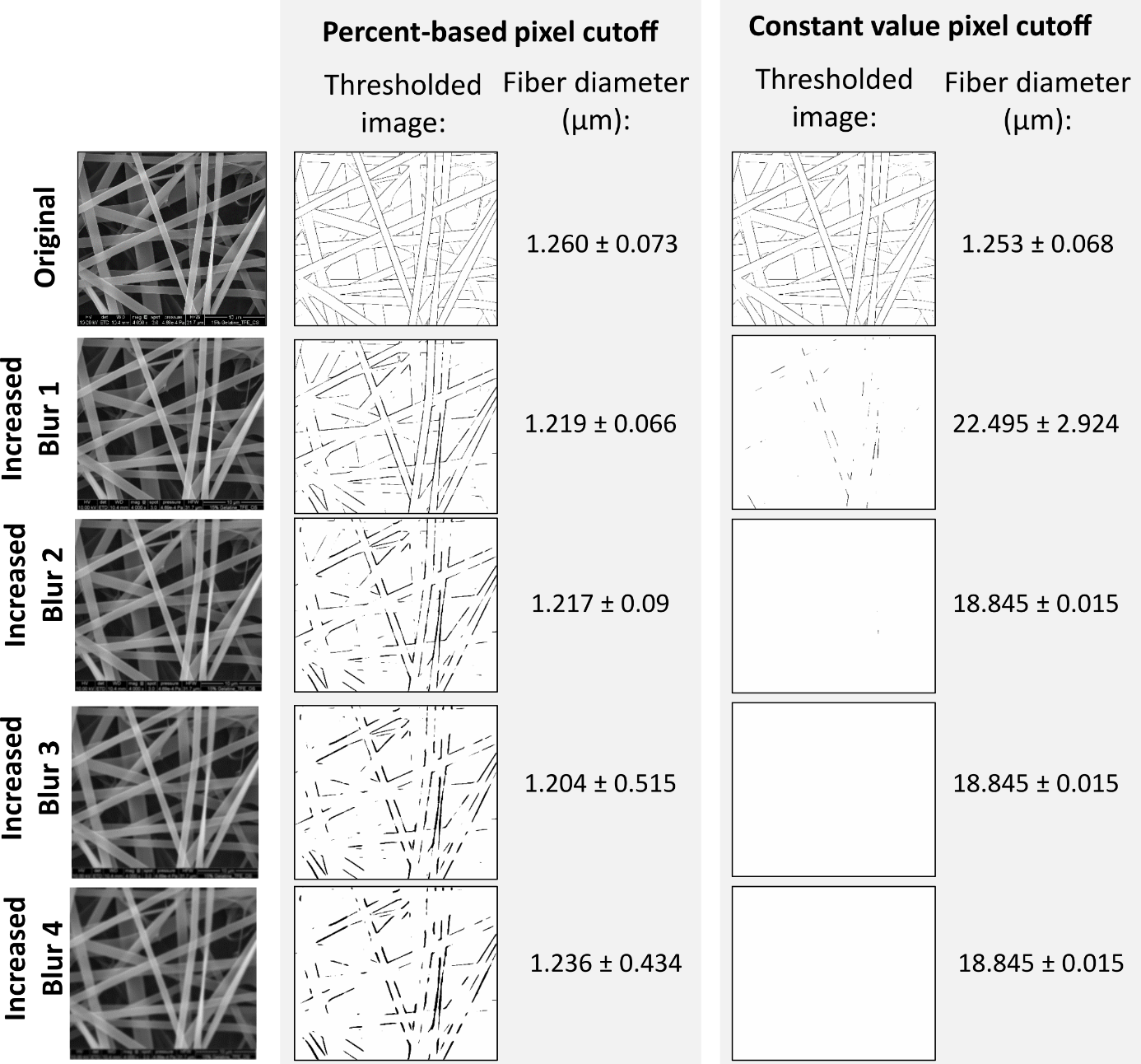


**S2 Fig. Testing thresholding methods with blurry images**. A set of test images was made using the 15% gelatin SEM fiber image. The original image was adjusted in ImageJ to be increasingly blurry using the Gaussian Blur function. These blurred images were then analyzed with the GIFT macro using either percent-based thresholding or a constant thresholding value. For the percent based analysis, the threshold cutoff was determined so that 5% of darkest pixels remained after thresholding and this was calculated for each image. For the constant value thresholding analysis, a cutoff grayscale value of 200 was used for all images. The original images are shown in the left-hand column. The results of the thresholding image analysis step and the resulting average fiber diameter measured in each case are shown for both thresholding methods. The percent-based method is more consistent when presented with a batch of images with blurry edges.

**
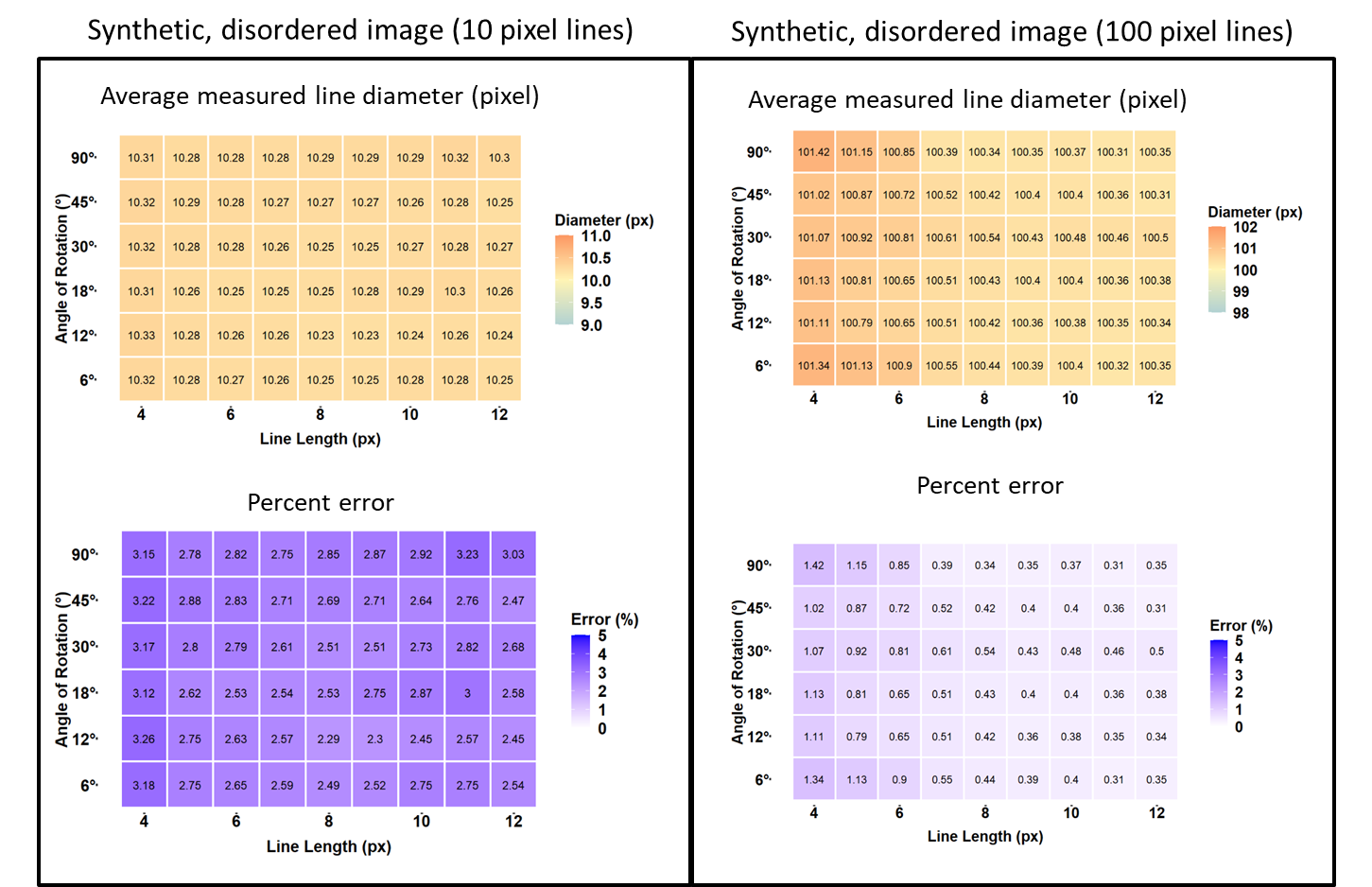
**

**S3 Fig. Synthetic image parameter sensitivity.** Disorder synthetic images with lines of 10 or 100 pixel diameter were analyzed using the GIFT macro with all combinations of 90, 45, 30, 18, 12 or 6° angles of rotation and 4-12 pixel line length. A 1% threshold was set because the synthetic images contain only black and white pixels. The top row of graphs showing the resulting fiber diameter measurements were graphed as a heat map to visualize the sensitivity of GIFT to changes in parameters when measuring a line with a known diameter. In the graphs of line diameter, the diameter measured with default parameters was set as the midpoint of the scale bar (yellow color). Average diameters higher than the default are represented by increasingly red hues and diameter results lower than the default are increasingly blue in color. The actual measured value is written on each point. The bottom row of graphs shows the percent error based on the known diameter of the lines at each combination of parameters.


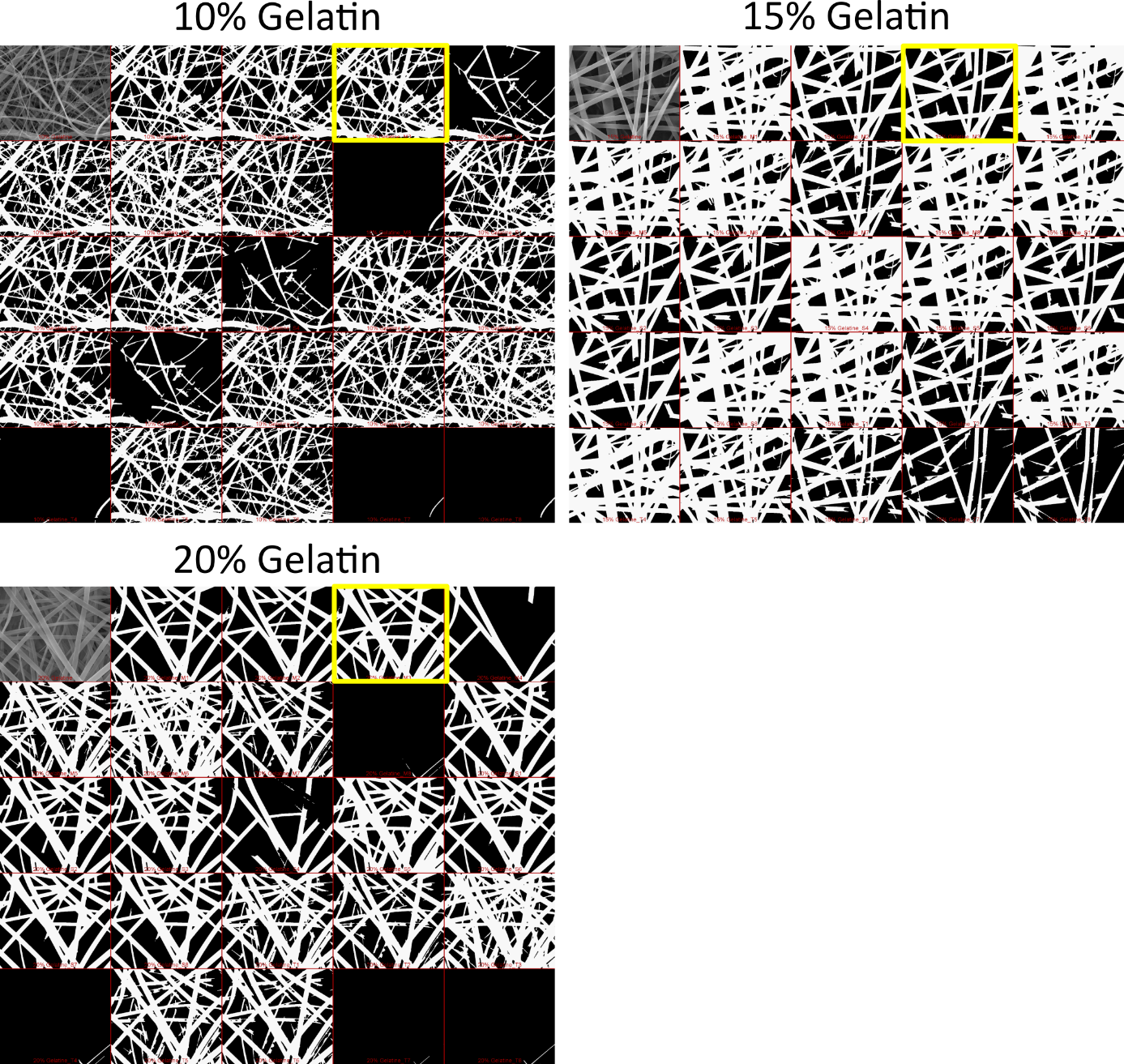


**S4 Fig. Segmentation images from DiameterJ.** During analysis, DiameterJ runs 24 segmentation methods and requires the user to select the best method before proceeding with diameter measurement. The montage of all segmentation images created by DiameterJ is shown here for the three analyzed SEM images of gelatin fibers. The top left image in each case is the original image and the selected segmentation method (M3) for this study is highlighted in yellow. Significant differences between segmentation methods can be seen.


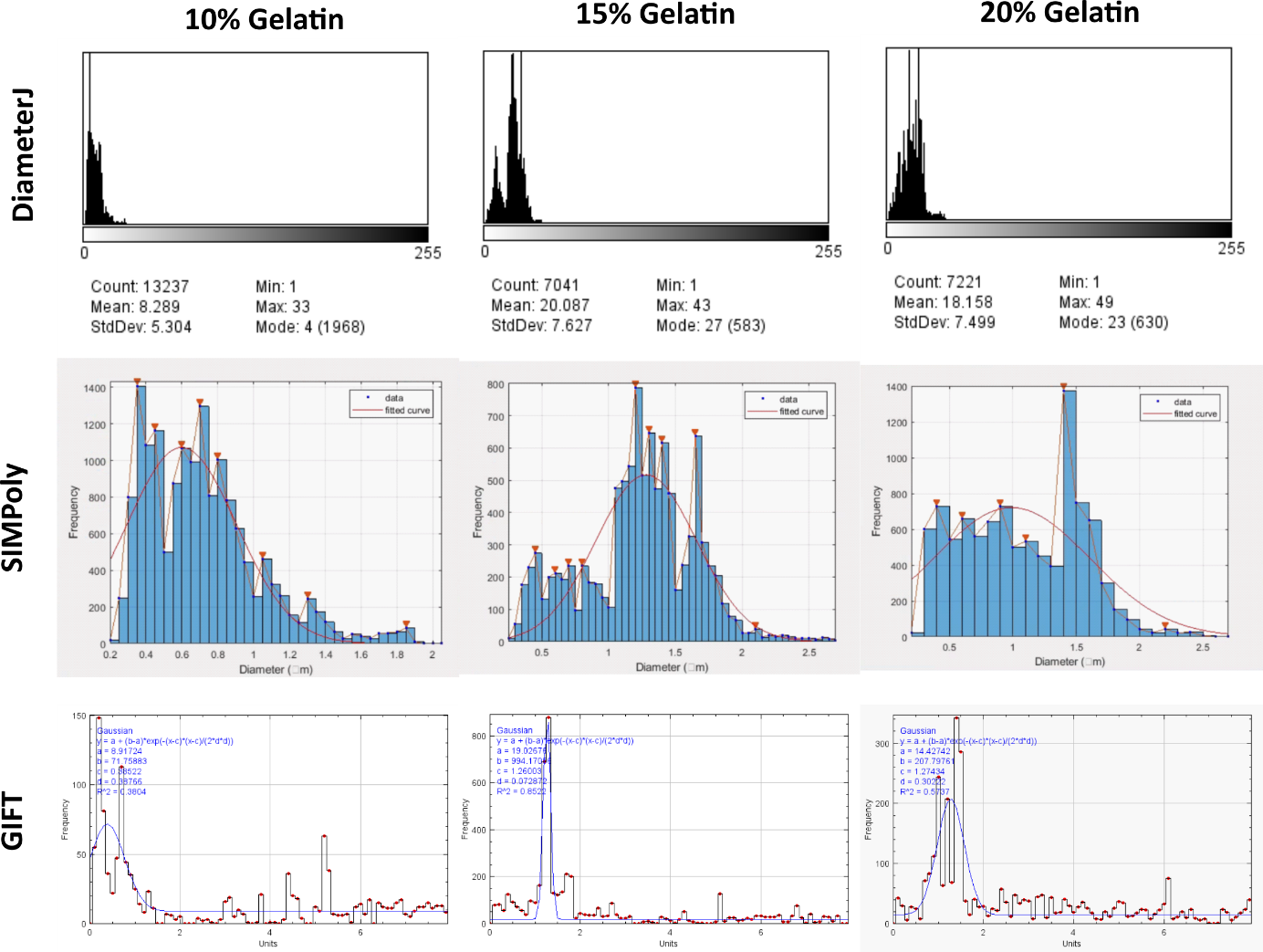


**S5 Fig. Histograms from all tested software.** DiameterJ, SIMPoly and GIFT all create histograms based on fiber width measurements and then use a Gaussian fit to determine a mean fiber diameter. This figure shows the histogram generated for the three SEM images of electrospun gelatin fibers for direct comparison. The DiameterJ graphs shown measurements in pixels, the measurements represent the fiber radii and the results are based on the results from the M3 segmentation method. The SIMPoly and GIFT graphs show measurements in µm based on the provided scale and default analysis parameters.
